# Clinicopathological Considerations in the Treatment of Unicystic Adenoid Ameloblastoma in Young Adults: A Clinical Case

**DOI:** 10.7759/cureus.87753

**Published:** 2025-07-11

**Authors:** Uma V Datar, Mamata Kamat, Sanjay S Byakodi, Karishma M Desai

**Affiliations:** 1 Oral Pathology and Microbiology, Bharati Vidyapeeth (Deemed to be University) Dental College and Hospital, Sangli, IND; 2 Oral and Maxillofacial Surgery, Bharati Vidyapeeth (Deemed to be University) Dental College and Hospital, Sangli, IND; 3 Oral and Maxillofacial Pathology, Tokyo Dental College, Tokyo, JPN

**Keywords:** adenoid ameloblastoma, ameloblastoma, conservative treatment, odontogenic tumour, unicystic ameloblastoma, unicystic variant, who 2022 classification

## Abstract

This article presents the case study of a 26-year-old female patient who came to our institute with pain and swelling in the left side of her lower jaw and face that had persisted for over a month. A histological examination of the lesion revealed a cystic odontogenic epithelium with a flat interface between the epithelium and connective tissue. This was accompanied by juxtaepithelial induction and mural proliferation in the form of follicles. The tumour epithelium met the Vickers-Gorlin criteria, and in some areas, the tumour cells were arranged in a plexiform pattern. We also observed regions of degeneration, a dentinoid component, and cells organised in a whorled pattern associated with the dentinoid. The lesion was diagnosed as unicystic adenoid ameloblastoma (UAA). After conservative treatment, the lesion recurred one year later, showing histological features similar to those of the primary lesion. UAA requires meticulous clinical and pathological examination, as well as a tailored management approach, to prevent recurrence and achieve optimal treatment outcomes.

## Introduction

Adenoid ameloblastoma (AA) has been recognised as a separate entity in the 2022 World Health Organization (WHO) classification of odontogenic tumours [1]. AA shows odontogenic epithelium in a plexiform arrangement or as follicles, with peripheral cells exhibiting ameloblast-like differentiation and central areas with adenomatoid changes, including pseudo-ducts and whorled structures [2]. There is considerable overlap between the histological features of AA and those of ameloblastoma, adenomatoid odontogenic tumour (AOT), and other odontogenic tumours [3]. There may be underreporting of AA cases due to challenges in diagnosis and the previous lack of recognition of this tumour as a distinct entity. To date, approximately 50 cases of AA have been reported [4-9]. A few cases of its peripheral and unicystic variants have been reported since its inclusion as a separate entity [10,11].

Ameloblastoma develops through the aberrant activation of signalling pathways involved in tooth formation. Conventional ameloblastomas primarily exhibit mitogen-activated protein kinase (MAPK) pathway activation, particularly through BRAF V600E/FGFR/SMO, promoting odontogenic epithelial proliferation and local invasiveness [3,4]. In contrast, AA (recognised in the 2022 WHO classification) displays distinct molecular behaviour, with the absence of BRAF and KRAS mutations, and instead shows WNT pathway dysregulation, commonly through CTNNB1 mutations and nuclear β-catenin localisation [4,6].

Given its unique behaviour, higher recurrence potential, and molecular distinction, AA warrants separate diagnostic consideration. This report discusses a case of recurrent ameloblastoma with a unicystic histological pattern, thus diagnosed as unicystic adenoid ameloblastoma (UAA), in a young female patient. Herein, we also discuss challenges in management and review the literature and its clinical implications.

## Case presentation

**Figure 1:**
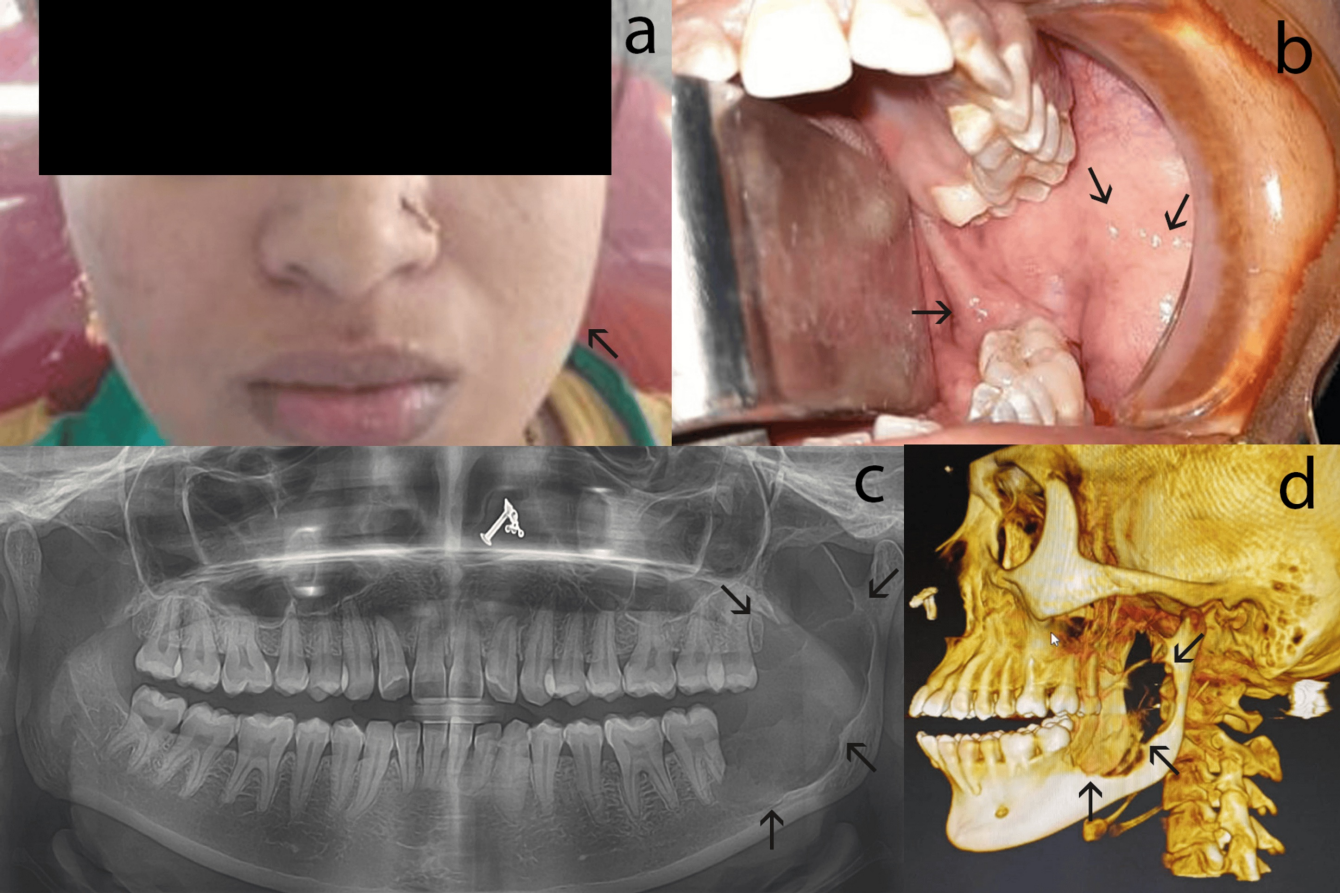
Clinical and radiographic findings of this case (a) An extraoral photograph showing a slight swelling in the mandibular left posterior region. (b) An intraoral photograph displaying the affected region. (c) An OPG revealing multilocular radiolucency that extends from tooth 37 to the ramus. (d) A 3D CBCT reconstruction showing the involvement of the left mandible and ramus of the mandible (arrows pointing to the lesion). OPG: orthopantomograph; CBCT: cone-beam computed tomography

A 26-year-old female patient presented with pain and swelling in the left lower jaw for one month. She had undergone an extraction of the lower left third molar three months ago. Clinical examination revealed a 4 × 4 cm swelling in the mandibular left posterior region. This swelling involved the angle of the mandible and extended up to the ramus of the mandible (Figure 1a, 1b). Radiographic evaluation revealed a large, well-defined radiolucent lesion with scalloped margins extending from the area of 37 to the coronoid process of the mandible; the distal root of 37 was resorbed (Figure 1c, 1d).

Considering the patient's age and clinical and radiographic presentation, enucleation of the lesion followed by reconstruction was planned. The surgical procedure was performed under general anaesthesia via nasotracheal intubation with the patient in supine position and her head turned to the right to allow optimal access to the left mandibular region. Under aseptic conditions, a crevicular incision was made from teeth 33 to 38 with a posterior release. A full-thickness mucoperiosteal flap was raised, and the cortical bone was removed to reveal the cystic lesion extending from the posterior mandibular body to the ramus, coronoid process, and sigmoid notch. The cyst was enucleated in total using blunt dissection. Tooth 37 exhibited resorption of the distal root and was therefore extracted. The cavity was debrided and irrigated, and the site was closed with 3-0 resorbable sutures after achieving haemostasis. The specimen was sent for histopathological evaluation.

**Figure 2:**
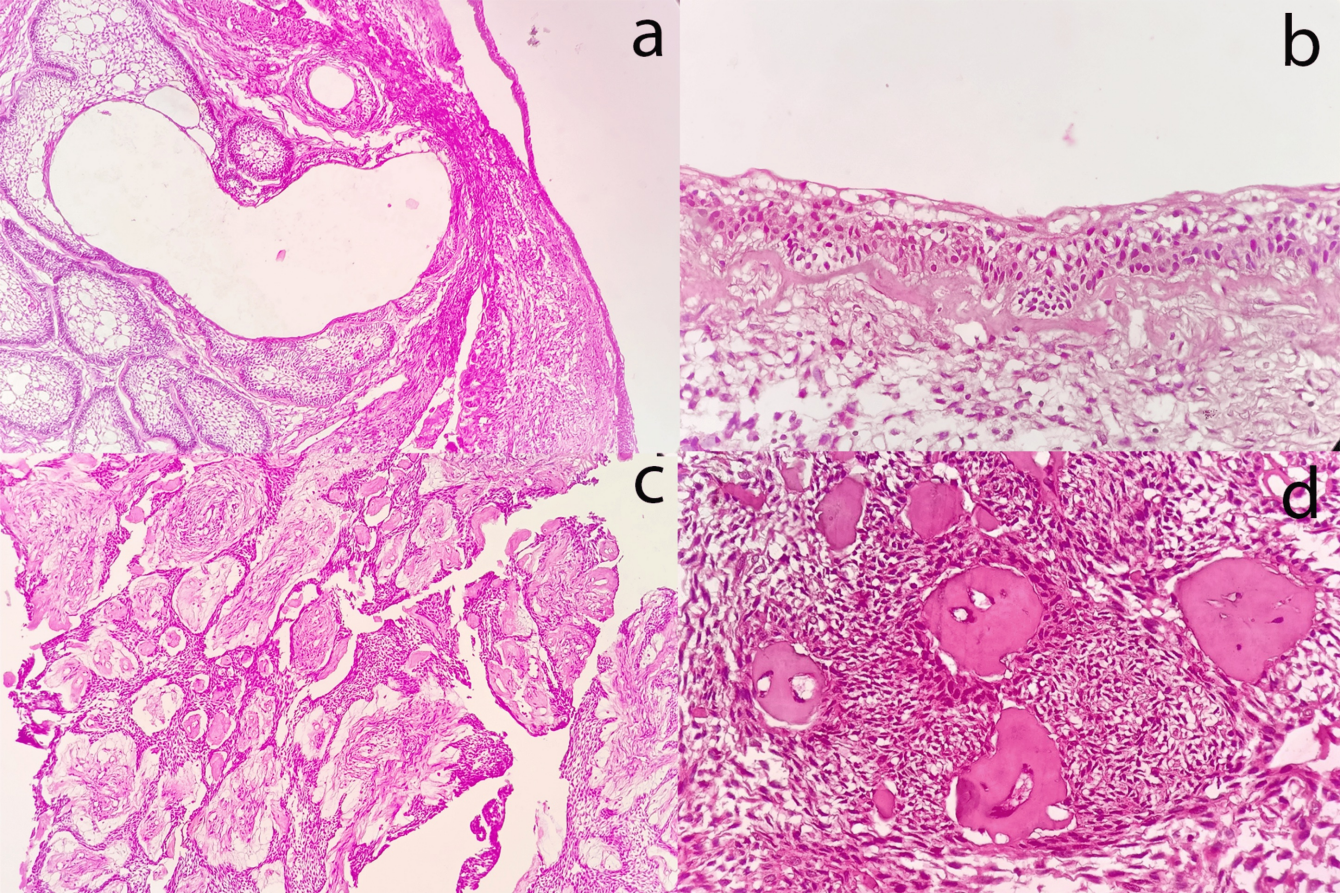
Histopathological features of this case (a) Unicystic ameloblastoma exhibiting mural proliferation, with small cyst formations observed within the islands (H&E: 4×). (b) The higher-magnification image reveals tumour cells in the cystic lining, characterised by tall columnar cells that display reversal of polarity and subnucleolar vacuoles (H&E: 10×). (c) The tumour cells are arranged in a cribriform pattern (H&E: 4×). (d) Dentinoid tissue is present, surrounded by tumour cells (H&E: 10×). H&E: hematoxylin and eosin

Histologically, the enucleated tissue showed a lumen that was lined by a 3-4-cell-layer-thick odontogenic epithelium; the follicles in the connective tissue exhibited peripheral tall columnar cells, reversal of polarity, stellate reticulum-like cells, cystic degeneration within the follicles, and induction surrounding the follicles (Figure 2a, 2b). Plexiform pattern with degeneration, solid cribriform areas (Figure 2c), and spindled whorls with eosinophilic dentinoid material (Figure 2d) were also noted.

**Figure 3:**
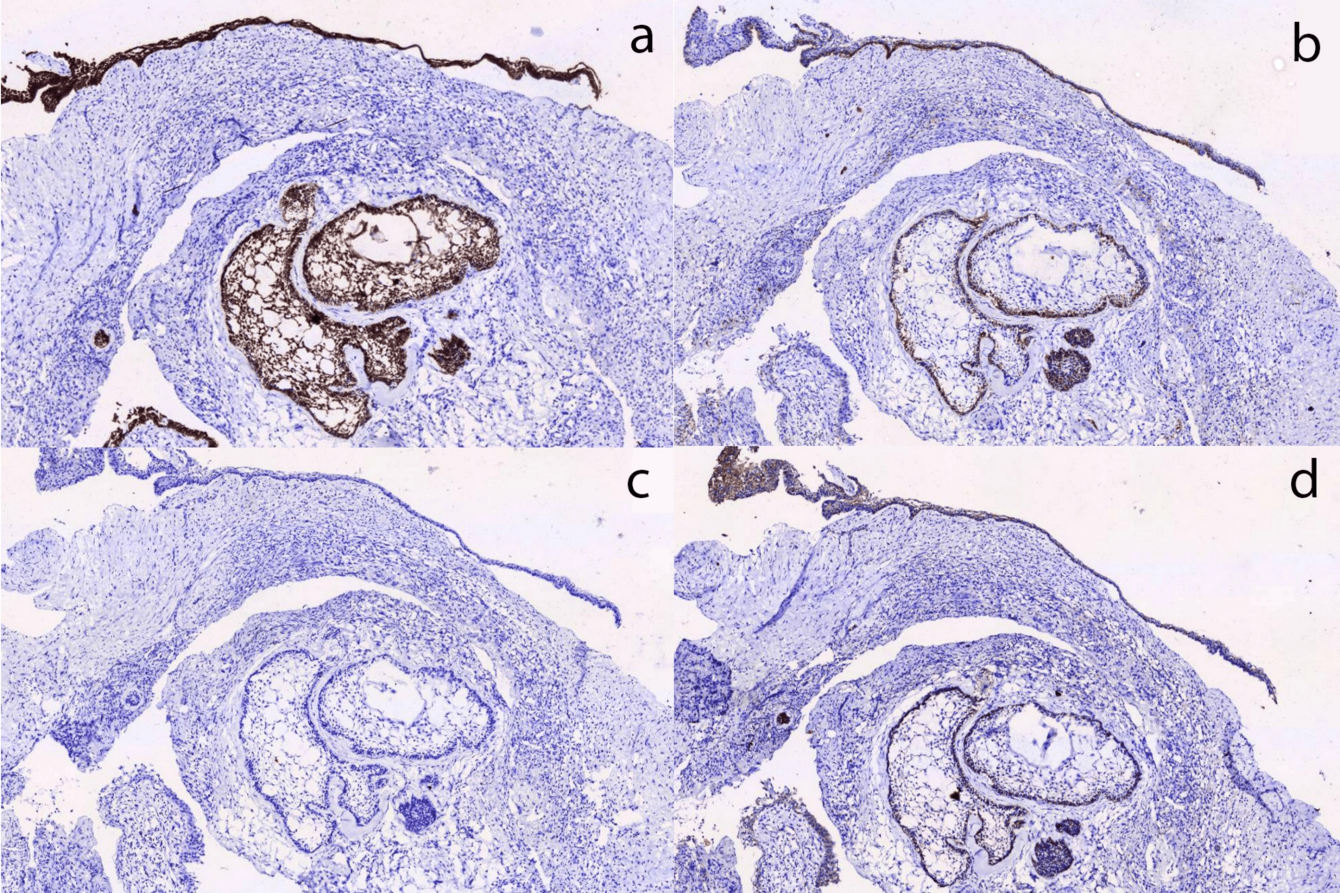
Immunohistochemical findings of this case Photomicrograph of tumour cells showing (a) strong positivity for CK19, (b) positivity for podoplanin, (c) weak reactivity to Ki-67, and (d) weak reactivity to β-catenin.

Immunohistochemical analysis revealed strong expression of CK19 and podoplanin, moderate expression of β-catenin, and least expression of Ki-67 (Figure 3a-3d). 

**Figure 4:**
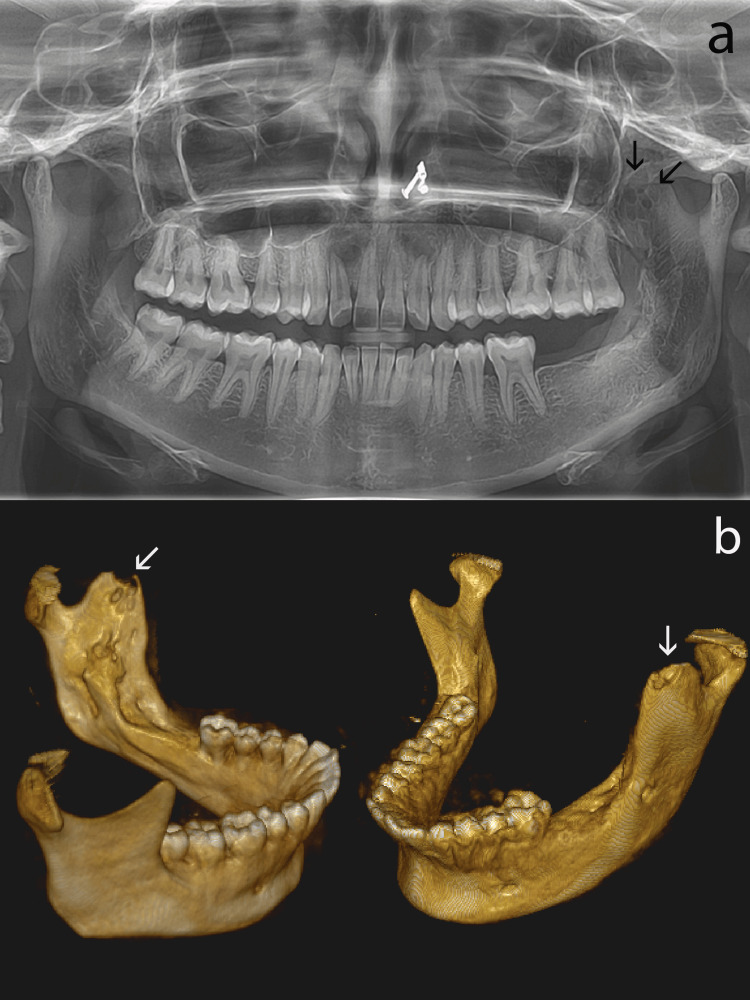
Radiographic and histopathological findings of the recurrent lesion (a) A one-year follow-up OPG reveals a multilocular radiolucency affecting the coronoid process (black arrows indicate the multilocular lesion in the coronoid process). (b) A 3D CBCT reconstruction shows partially healed tissue in the left body of the mandible and ramus, along with a defect in the coronoid process (white arrows point toward the lesion involving the coronoid process). OPG: orthopantomograph; CBCT: cone-beam computed tomography

After a year of follow-up, radiographic examination revealed a recurrence in the coronoid process, as illustrated in Figure 4a, 4b.

**Figure 5:**
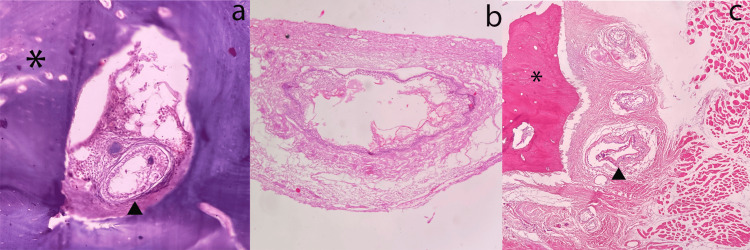
Histological findings of the recurrent lesion (a) The tumour island is observed in association with bone. (b) The recurrent tumour presented in a unilocular pattern. (c) The tumour is invading muscle tissue (asterisks indicate bone tissue; arrowheads point to the tumour islands).

Considering the age of the patient and early recurrence, a coronoidectomy was performed. The biopsy of the recurrent lesion (Figure 5a-5c) was consistent with the previous diagnosis of UAA. 

After the coronoidectomy, the lesion recurred again after one and a half years, necessitating a hemimandibulectomy.

## Discussion

UAA is a less frequently encountered subtype of the newly recognised entity AA. While AA is characterised by a combination of AM and AOT features, the unicystic variant presents with a distinct cystic component. The unicystic presentation of AA is gaining attention due to its innocuous clinicopathological features and contrasting biological behaviour. Martins-Chaves et al. first reported UAA and retrospectively identified four more cases [11-15]. Additionally, Sachdev et al. conducted a systematic review, identifying two cases of AA with a well-defined unicystic component [5,16,17]. These cases have been compiled and summarised in Table 1.

**Table 1:** Summary of cases of UAA NP: not provided; AOT: adenoid odontogenic tumour; AA: adenoid ameloblastoma; COC: calcifying odontogenic cyst; UA: unicystic ameloblastoma; UAA: unicystic adenoid ameloblastoma

Author	Age	Duration	Site	Radiographic findings	Histopathological diagnosis	Treatment (first)	Recurrence
Tajima et al. (1992) [13]	35/M	NP	Anterior mandible	Unilocular radiolucency with focal radiopacity	Ameloblastoma arising in COC	Surgical resection	No recurrence during the five-year follow-up
Evans et al. (2004) [14]	39/M	1 year	Anterior mandible	Partial radiolucency	AOT	Enucleation	Three recurrences
Jivan et al. (2007) [16]	40/M	7 months	Mandible	Unilocular radiolucency	AOT arising in UA	NP	NP
Ide et al. (2009) [12]	44/M	NP	Anterior maxilla	Unilocular radiolucency	AA with dentinoid	Enucleation	Four recurrences
Sathyanarayana et al. (2017) [17]	16/F	2 months	Mandible	Unilocular radiolucency	AOT with UA	Surgical resection	Five recurrences
Rai et al. (2017) [15]	55/M	3 months	Right mandible	Unilocular radiolucency	AA with dentinoid	Enucleation	NP
Martins-Chaves et al. (2022) [11]	34/M	72 months	Maxillary	Unilocular radiolucency	UAA	Enucleation	NP
Present case	26/F	1 week	Left mandible	Multilocular radiolucency	UAA	Enucleation	Two recurrences

UAA shows male predominance and presents frequently in the fourth decade of life. It presents as a well-defined unicystic lesion [2]. The unicystic variant of conventional ameloblastoma may present as a multilocular lesion [18]. Likewise, the UAA presented as a multilocular lesion in the present case. Since the histological features of UAA overlap with those of ameloblastoma, AOT, and other odontogenic tumours, a significant number of UAA have been misdiagnosed initially as other odontogenic tumours (Table 1). This has often led to multiple recurrences necessitating multiple surgical interventions.

UAA is characterised by the same histopathological features that characterise AA, including the cellular arrangement pattern of odontogenic epithelium, peripheral ameloblastic-like cells, adenomatoid changes, and dentinoid deposition [2]. Jayasooriya et al. reported that 70.8% of the AA presented as plexiform ameloblastomas, while duct-like and glandular structures typical of AOT were observed in 95.9% of AA [8]. These histopathological features support the classification of AA as a distinct entity.

AA is characterised by the expression of CK14, CK19, p40, p16, and p53. The Ki-67 proliferation index is reported to be high, which explains the locally aggressive behaviour and high recurrence rate (45.5-70%) [4,7]. However, a few cases have also shown low Ki-67 expressions, like the present case. Despite a low Ki-67 index, the lesion recurred in our case, possibly due to high podoplanin expression and conservative management.

The morbidity associated with AA stems from its locally aggressive nature and high recurrence rate, 45.5-70%, especially with conservative treatment [4,5,7,8]. UAA also shows considerable morbidity due to a high recurrence rate; five out of eight lesions have recurred, as depicted in Table 1. This implies a need for potentially extensive surgical interventions, follow-up, and management of recurrences, which can significantly impact a patient's quality of life and oral health. The occurrence of disease-related mortality has not been reported yet.

The crucial distinction between conventional ameloblastoma and its unicystic variant, and between AA and UAA, can be drawn through histological and immunohistochemical investigations. UAA's potential aggressiveness contrasts with its innocuous unicystic presentation. UAA and AA are more aggressive than conventional ameloblastoma. This is evidenced by a higher recurrence rate observed in AA compared to conventional ameloblastoma [7], as well as the high recurrence rate in the series of UAA (Table 1) when a conservative treatment approach was employed. More follow-up data is needed to determine the long-term prognosis of conservative treatment of UAA. On appraising the evolving understanding of this newly identified entity, we opine that though a conservative approach is desirable in young patients, the extent of surgical excision must be determined after a thorough assessment of the immunoprofile and the radiographic extent of the lesion. Accurate diagnosis and appropriate management are a mainstay of successful treatment outcomes.

## Conclusions

UAA is a rare odontogenic tumour with a complex clinicopathological profile. Recent evidence suggests that there is a tendency for recurrence after conservative treatment. Unlike the significant behavioural differences between conventional solid and unicystic ameloblastoma, a UAA does not necessarily indicate a less aggressive behaviour. Considering the risk of misdiagnosis with less aggressive entities such as AOT or conventional unicystic ameloblastoma, meticulous histological evaluation is paramount for accurate diagnosis. Besides the intrinsic aggressive nature of AA, key factors to consider when diagnosing and managing UAA include immunoprofile, mural invasion, and radiographic features. Compared to other odontogenic tumours, UAA appears to have a higher recurrence rate when treated conservatively; however, more research is needed for definitive comparisons. The multiple recurrences emphasise the importance of early recognition, surgical resection with wide margins, and long-term surveillance in these cases.
